# Supplementary material for: Building a community of practice through social media using the hashtag #neoEBM
Source: PLoS One. 2021 May 28;16(5):e0252472. doi: 10.1371/journal.pone.0252472 (PMC8162580; doi:10.1371/journal.pone.0252472)
Supplement: Attachment — Submitted filename: Reviewer-plos one.docx [file pone.0252472.s002.docx]

**Reviewer comments :**

Building a community of practice through social media

The study was interesting to go through. These are my main concerns.  Below comments follow the order of the manuscript.

**Title**

I recommend adding to the title as it is not clear enough (too broad).
 **Introduction**
The authors should add to the introduction a paragraph on the importance of the practice and gap in knowledge. Also, they need to add some literature in this regard. Other comments in introduction part is indicated as below:

Line 65: the authors should change the numbering of the reference to number 5 into 2, so it will follow sequential order.

Line 70: please delete the word meeting, as it’s a repetition.

Line 73: start a new Paragraph-Twitter is an….

Line 78: Please clarify the type of the observational study conducted.

**Material and Methods**
-Lines 83-85: the study design should be clarified.

-Sample size and sample size calculation.

-Where there any exclusion criteria?

**Results**

Figure 1: the writing in the figure is not clear.

Table 1: Aveling’score- please edit the space between s c.

**Discussion**
-This should be supported by other studies in different medical fields that show an effect of social media on Cop.

-Need recommendations for further studies and what should be done.
 **Thank you**
