# Supplementary material for: Building a community of practice through social media using the hashtag #neoEBM
Source: PLoS One. 2021 May 28;16(5):e0252472. doi: 10.1371/journal.pone.0252472 (PMC8162580; doi:10.1371/journal.pone.0252472)
Supplement: Attachment — Submitted filename: Response_To_Reviewers_FINAL.docx [file pone.0252472.s003.docx]

**Thank you to the Editorial Office, Editor and Reviewers for their invaluable comments. Please find our detailed responses below:**

**Journal Requirements:**

When submitting your revision, we need you to address these additional requirements.

**Journal Requirements:**

**Comment 1**. Please ensure that your manuscript meets PLOS ONE's style requirements, including those for file naming. The PLOS ONE style templates can be found at

<https://journals.plos.org/plosone/s/file?id=wjVg/PLOSOne_formatting_sample_main_body.pdf> and

<https://journals.plos.org/plosone/s/file?id=ba62/PLOSOne_formatting_sample_title_authors_affiliations.pdf>

**Reply**: We have carefully reviewed the above documents and made the changes accordingly in our revised paper.

**Comment 2**. In your Methods section, please include additional information about your dataset and ensure that you have included a statement specifying whether the collection method complied with the terms and conditions for the website.

**Reply:** This is now included in the Methods section.

**Comment 3**. In your ethics statement in the Methods section and in the online submission form, please confirm that all data were fully anonymized before you accessed them and/or whether the IRB or ethics committee waived the requirement for informed consent.

**Reply**: We have added this sentence to our methods as well as to the online form.

“The requirement for individual consent was waived by the HREC.”

**Comment 4**. Thank you for stating the following in the Competing Interests section:

"AK, RP, CW, SM, AS, OE and BK are current or previous social media editors and NB is immediate past lead social media editor for the International Society for Evidence-Based Neonatology ([ebneo.org](http://ebneo.org)) and all tweet as part of these roles as @EBNEO. None are compensated for their role as social media editors. The authors have no financial relationships with the online analytical platform Symplur."

Please confirm that this does not alter your adherence to all PLOS ONE policies on sharing data and materials, by including the following statement: "This does not alter our adherence to  PLOS ONE policies on sharing data and materials.” (as detailed online in our guide for authors http://journals.plos.org/plosone/s/competing-interests).  If there are restrictions on sharing of data and/or materials, please state these. Please note that we cannot proceed with consideration of your article until this information has been declared. Please include your updated Competing Interests statement in your cover letter; we will change the online submission form on your behalf.

**Reply**: We have now included in the Completing Interests section in our cover letter and thank you in advance for updating the online submission form for us

Please know it is PLOS ONE policy for corresponding authors to declare, on behalf of all authors, all potential competing interests for the purposes of transparency. PLOS defines a competing interest as anything that interferes with, or could reasonably be perceived as interfering with, the full and objective presentation, peer review, editorial decision-making, or publication of research or non-research articles submitted to one of the journals. Competing interests can be financial or non-financial, professional, or personal. Competing interests can arise in relationship to an organization or another person. Please follow this link to our website for more details on competing interests: <http://journals.plos.org/plosone/s/competing-interests>

**Reply**: Thank you and noted.

**Comment 5**. We note that you have indicated that data from this study are available upon request. PLOS only allows data to be available upon request if there are legal or ethical restrictions on sharing data publicly. For information on unacceptable data access restrictions, please see <http://journals.plos.org/plosone/s/data-availability#loc-unacceptable-data-access-restrictions>.

**In your revised cover letter, please address the following prompts:**

a) If there are ethical or legal restrictions on sharing a de-identified data set, please explain them in detail (e.g., data contain potentially identifying or sensitive patient information) and who has imposed them (e.g., an ethics committee). Please also provide contact information for a data access committee, ethics committee, or other institutional body to which data requests may be sent.

b) If there are no restrictions, please upload the minimal anonymized data set necessary to replicate your study findings as either Supporting Information files or to a stable, public repository and provide us with the relevant URLs, DOIs, or accession numbers. Please see <http://www.bmj.com/content/340/bmj.c181.long> for guidelines on how to de-identify and prepare clinical data for publication. For a list of acceptable repositories, please see <http://journals.plos.org/plosone/s/data-availability#loc-recommended-repositories>.

We will update your Data Availability statement on your behalf to reflect the information you provide.

**Reply**: We have uploaded as Supplementary Information a minimal de-identified data set used to inform our paper. Additional data underlying the results presented in the study are available from the Symplur Signals database and can be accessed through www.symplur.com with a fee-based account subscribed for the hashtag #neoEBM. We paid US$499 for access to the database for one month through the Symplur Signals Self-Serve (research option for 10 datasets) option.

This dataset is also available here:

Keir, Amy (2021): Raw data on use on #neoEBM on Twitter: 2018-2021. The University of Adelaide. Dataset. <https://doi.org/10.25909/14329754.v1>

This information has been made clear in our Cover Letter.

**Reviewers' comments:**

**Comments to the Author**
1. Is the manuscript technically sound, and do the data support the conclusions?
The manuscript must describe a technically sound piece of scientific research with data that supports the conclusions. Experiments must have been conducted rigorously, with appropriate controls, replication, and sample sizes. The conclusions must be drawn appropriately based on the data presented.

Reviewer #1: Yes

Reviewer #2: Partly

Reviewer #3: No

Reviewer #4: Yes

2. Has the statistical analysis been performed appropriately and rigorously?

Reviewer #1: Yes

Reviewer #2: No

Reviewer #3: No

Reviewer #4: Yes

3. Have the authors made all data underlying the findings in their manuscript fully available?
The [PLOS Data policy](http://www.plosone.org/static/policies.action#sharing) requires authors to make all data underlying the findings described in their manuscript fully available without restriction, with rare exception (please refer to the Data Availability Statement in the manuscript PDF file). The data should be provided as part of the manuscript or its supporting information, or deposited to a public repository. For example, in addition to summary statistics, the data points behind means, medians and variance measures should be available. If there are restrictions on publicly sharing data—e.g. participant privacy or use of data from a third party—those must be specified.

Reviewer #1: Yes

Reviewer #2: Yes

Reviewer #3: No

Reviewer #4: No

4. Is the manuscript presented in an intelligible fashion and written in standard English?
PLOS ONE does not copyedit accepted manuscripts, so the language in submitted articles must be clear, correct, and unambiguous. Any typographical or grammatical errors should be corrected at revision, so please note any specific errors here.

Reviewer #1: Yes

Reviewer #2: No

Reviewer #3: Yes

Reviewer #4: Yes

**5. Review Comments to the Author**
Please use the space provided to explain your answers to the questions above. You may also include additional comments for the author, including concerns about dual publication, research ethics, or publication ethics. (Please upload your review as an attachment if it exceeds 20,000 characters).

**Thank you again for the detailed and very helpful comments from all the reviewers. Please find our detailed responses below:**

**Reviewer #1**: Reviewer comments:
Building a community of practice through social media. The study was interesting to go through. These are my main concerns. Below comments follow the order of the manuscript.

**Comment 1:**
Title
I recommend adding to the title as it is not clear enough (too broad).
**Reply:** We have altered the title to “Building a community of practice through social media using the hashtag #neoEBM”

**Comment 2:**
Introduction
The authors should add to the introduction a paragraph on the importance of the practice and gap in knowledge. Also, they need to add some literature in this regard.

**Reply:** Additional information about the evidence-to-practice gap is included now and referenced.

**Comment 3:**

Other comments in introduction part is indicated as below:
Line 65: the authors should change the numbering of the reference to number 5 into 2, so it will follow sequential order.

**Reply**: There was a referencing error and reference 5 has been corrected.
Line 70: please delete the word meeting, as it’s a repetition.

**Reply**: Deleted
Line 73: start a new Paragraph-Twitter is an….
**Reply:** New paragraph started.

**Comment 4:**

Line 78: Please clarify the type of the observational study conducted.
**Reply:** Cohort now included.

**Comment 5:**
Material and Methods
-Lines 83-85: the study design should be clarified.
-Sample size and sample size calculation.
-Where there any exclusion criteria?
**Reply:** We have included the following information in the Material and Methods section:

Our study was a descriptive, observational cohort study utilising the social media health analytic website Symplur as the data source. User generated posts (“tweets”) met inclusion criteria if they were generated on Twitter and contained the hashtag #neoEBM between 1 May 2018 and 9 January 2021. All other tweets were excluded. This was a convenience sample of all eligible posts without formal sample size calculation.

**Comment 6:**
Results
Figure 1: the writing in the figure is not clear.

Table 1: Aveling’score- please edit the space between s c.
**Reply:** Our apologies, the figure has been updated and the typographical error corrected.

**Comment 7:**
Discussion
-This should be supported by other studies in different medical fields that show an effect of social media on Cop.

**Reply:** Thank you for this comment, we have added further information and relevant citations into the discussion:

“Our findings are supported by the development of other online CoPs in healthcare.^7^^,^ ^8^”

**Comment 8:**
-Need recommendations for further studies and what should be done.
**Reply:** “While we have demonstrated elements of organisation, the impact of the CoP on the practice of individual clinicians is yet to be determined. Further qualitative and quantitative study is needed to understand how practice may change through participation in the #neoEBM community. It cannot also be assumed that all CoP will add value in the same way and further research is needed in different specialties and professions to see how widespread this approach is in healthcare.”

**Reviewer #2:** This is an article discussing on the use of social media to develop a community of practice. I appreciate the initiatives on such social media-based research and saw merits of this paper and should be considered for publication upon major corrections.

**My comments and suggestions**: The authors shall improve the manuscripts (also to serve the need of readers of PLOS ONE of different backgrounds, unlike medical journal of specialised readers). In brief, the manuscript needs to be better organised and provide further elaboration to support the ideas put forward in the manuscript as in the following areas:

**Comment 1:**
Introduction:
The numbering of the reference is not according to the sequence.

**Reply:** Thank you for this comment, we have corrected our referencing.

**Comment 2:** Perhaps, also some background on why or the driver of online CoP, perhaps from other established fields.
**Reply:** We have now included the following in the introduction/background:

The timely implementation of medical research evidence into clinical practice is an important public health challenge, with the evidence-to-practice gap famously described as taking up to 17 years.^2^ New approaches to narrow this gap are urgently needed, and online CoPs may be a valuable tool in an era of growing digital interaction.

**Comment 3:**
Materials and methods:
Some narration on NOECO by Roland et al shall be included to make your methods clearer.
**Reply:** We have now included a brief explanation around NOECO in the methods section.

**Comment 4:**
Results:
I feel that all data reported by authors should be analyzed and interpreted further, rather than to report as is. For example, the authors reported the cumulative users over time; however, data is only slightly described by the authors in the section of engagement metrics. Interpretation such as the trend of users and tweets, projection in future numbers, the number of users with different number of tweets could add values to the manuscript.

**Reply:** We appreciate the request for further detail in the analysis, but we are currently limited by information provided by third party platforms. We have provided information commensurate with previous research in this area (reference 8) and hope that this work will enable future research groups, including ours, to push to expand data sources to enhance the information available.

**Comment 5:**
I expect the information such as the locations and twitters can also be further described and interpreted, rather than simply reported the data. (For instance, rhe authors reported from selected countries with numbers, and what about the rest, since the readers will be curious to know, for instance, from a total of 3228, US, UK, Spain and Australia were 494, 467, 110 and 103, what about the other locations of the remaining 2054?)

**Reply:** We have added language to the Methods to clarify that the location of the user is only available when publicly shared by the user and updated to Results to describe the proportion of users lacking this information. Unfortunately, no further information of the location much beyond what is supplied in the paper is available – the majority of users’ locations are deemed ‘unknown’.

**Comment 6:**
Of course, the figures attached must be in good resolution, and the detailed information is difficult to read.

**Reply:** Please accept our apologies, we have updated the figures to a higher resolution and reviewed them closely for legibility. These are now formatted in TIFF format at 600 dpi per PLOS One guidance.

**Comment 7:**
Table 1 compared the core components of a CoP and the characteristics of community. This is the key finding that #neoEBM community fits into the 8 features by Aveling. The information in this table shall be further explained by the authors. For example, the top 20 twitter users were identified by the authors, and they were said to be originated from interdependent groups and individuals, and the members cross clinical and organizational boundaries. How does this determination made by the authors were not described and it should be supported by the background data?

**Reply:** Further detailed data on the individual Twitter users identified within this group is now provided in the Supplementary Information.

**Comment 8:**
As for the content (second row), the authors stated that the twitter users were members united for a common purpose and come to achieve the same aim, how do the authors come to such determination shall be discussed and supported by the analysis. The same also goes to other points in Table 1. A further description on the data will benefit the readers for better understanding.

**Reply:** We have included additional information in the Supplementary Material and ensured that there is a clear justification for each assertion in Table 1.

**Comment 9:**
Discussion:
There were several issues which need attention by the authors. How the authors determined the identity of twitter users, the issue of robotic tweets, potential of mis-hashtag by the users, confidentiality of the tweeted message, issue of the authorship on tweeted information, and etc are factors that potentially affect the interpretation of the data.

**Reply:**

Twitter users were identified by the use of the #neoEBM hashtag. It would be possible for some of the users to have been ‘bots’ however these numbers are likely to be small because (i) NeoEBM is a very niche hashtag and there is little or no commercial value and (ii) the authors of the study have lived experience in twitter and are able to recognise individuals outside of social media norms. Twitter is a public forum in which you sign an agreement at the outset that your tweets will be made public unless you specifically specify them to be private. Access to this data set means all tweets have been made publicly available. Finally, while it is possible that people were tweeting on behalf of other users this would have to have occurred at a massive scale to have impacted on the results, especially given the homogeneity shown in the content themes, and so we feel this is of limited concern.

**Comment 10:**
Also, I expect the authors to provide some view how the findings will lead to a greater quality improvement (QI) in clinical community in general.

**Reply:**

We hope that our work is a facilitator for the change processes needed at collective, organisational and cultural levels to improve the use of evidence in everyday clinical practice. We have briefly touched on and cited the following paper in the response to the reviewer’s comment:

Aveling EL, Martin G, Armstrong N, Banerjee J, Dixon-Woods M. Quality improvement through clinical communities: eight lessons for practice. J Health Organ Manag. 2012;26(2):158-74. doi: 10.1108/14777261211230754. PMID: 22856174.

**Comment 11:**
Reference:
The list needs some minor formatting.

**Reply:** Thank you, we have done this.

**Reviewer #3**:

In my opinion, the study titled “#neoEBM: Building a community practice through social media” is interesting but has major limitations:

**Comment 1:**
♣ Introduction: A clear rationale for the current study along with use of tools such as “Aveling’s core component of clinical community” is missing.

**Reply:** Thank you for this comment. We have more clearly written in the introduction that there is evidence (referenced) that highlights social media communities on twitter may act as communities of practice. There is therefore a theoretical link between the development of emerging communities of practice and improvements in care (although the extent of this link needs further evaluation). Communities of practice have previously not been described in neonatal medicine and this study sought to examine this.

**Comment 2:**
♣ Materials and methods: missing inclusion exclusion criteria for evaluating the dependent variables. This is vaguely presented but the actual process must be clearly presented.

**Reply**: We have included (in addition to more detail about the study design in response to reviewers’ other comments), the following:

“Our study was a descriptive, observational cohort study utilising the social media health analytic website Symplur as the data source. User generated posts (“tweets”) met inclusion criteria if they were generated on Twitter and contained the hashtag #neoEBM between 1 May 2018 and 9 January 2021. All other tweets were excluded. This was a convenience sample of all eligible posts without formal sample size calculation.”

**Comment 3:**
♣ The results section needs significant work. The figure axes are not legible. The quality of figure content is very poor. Additionally, the authors need to elaborate on the results and their relation to the presented figures.

**Reply**: The figures have been updated to be legible (again, please accept our apologies for this) and we have adjusted the axis labelling to improve legibility. These are formatted in TIFF format at 600 dpi per PLOS One guidance. Please also see response below.

**Comment 4:**
♣ Figure 2: it is expected that the frequency of tweets would be higher around scientific events as highlighted by the authors. But it is important that the authors elaborate on what this means and involves in their discussion.

**Reply:** Thank you for this comment and this demonstrates that our findings mimic real-life observations (thus validating our work) and we have included in the discussion the following “*highlight how communities of practice benefit from face-to-face interactions at conferences*.”

**Comment 5:**
♣ Discussion needs significant work on the above factors.

**Reply**: Please see our responses and additions to the paper in response to the above (and other significant additions to the discussion).

**Comment 6:**
♣ In the conclusion, the authors make a case that #neoEBM is an effective tool to disseminate research findings via social media platform (twitter in this case). What percentage of the research community would this reach? Are there any analyses to project the percentage of population it would reach compared to the standard Pubmed, google scholar and other scholar manager approaches? Is the information through #noEBM from twitter reliable? The significance and impact of these findings need to be discussed.

**Reply**: Further details regarding the additional limitations to our work has been added to the discussion. We have also included further information in the discussion around future research directions that hopefully also addresses this comment.

**Comment 7:**
♣ Finally, the data were not easily accessible to review.

**Reply**: We have included our basic dataset as Supplementary Information.

**Reviewer #4**:

Thank you for giving me the opportunity to review this paper. This paper addresses an interesting topic of building a community through Twitter, in order to increase the dissemination of research recommendation. The idea is great, the team had done a good job in building this community. However, several information was missing. The comments below are intended to further strengthen the paper.

**Comment 1:**
Title
1- The title could be improved by removing the hashtag, to be "Building a community of practice through social media".
**Reply:** Thank you for this comment. It does conflict with another reviewer’s comment to make the title more specific.

At this point we have altered it to state:

“Building a community of practice through social media using the hashtag #neoEBM”. However, we would be happy to alter it again as needed.

**Comment 2:**
Abstract
1- Line 57, can you please provide the long form of any abbreviation when you mention in it the first time, please?
**Reply:** We have removed the abbreviation and used the long-form.

**Comment 3:**
Introduction
1- It was not clear about the context in which the hashtag was introduced. Was there any previous attempt to engage people using for example, a mailing list? Did all the group twitter users get any formal training for using twitter when it was introduced? were there anyone in charge of the hashtag? How many hours per week was spent in observing the hashtag? Was there any previous attempt to assess the utilization of the hashtag?

**Reply:** The formation of #neoEBM was a spontaneous event by clinicians already engaged and knowledgeable about twitter. The reason this started was that the group were keen to more dissemination of research beyond email lists, and we were all familiar with twitter.

By definition, there is no one in charge of hashtag, as this is the way in which an organic community of practice develops. Some previous work on this has been published (reference 5: Keir et al, EBM_BMJ 2019).

**Comment 4:**
2- Line 65, can you please add the references numbers based on their appearance in the paper? As the introduction line 63 started with reference number 1, and line 65, the second appeared reference number is 5. Can you please clarify if this should be number 2?

**Reply:** Our apologies, there was an error in the referencing, and we have corrected it.

**Comment 5:**
3- Can you please provide a references to the statements in lines 74-76.

**Reply:** We have referenced these statements.

**Comment 6:**
4- Can you please provide a definition for the word hashtag, line 77.

**Reply:** We have provided a definition for the word hashtag accordingly.

**Comment 7:**
Materials and methods
1- Line 83, Can you please comment on the stage of hashtag registration please? Was this completed to assess the analysis using the Symplur software, or was this needed by Twitter, to register official hashtag?

**Reply:** We registered the hashtag #neoEBM within 3 days of our meeting at PAS in 2018 with the concept to monitor its usage over time using the Symplur software. Registration was required by Symplur to allow us to use their analytics platform.

**Comment 8:**
2- Line 85, can you please confirm what is NOECO, please?

**Reply:** Please see our reply to Reviewer 2, Comment 3. It is a standardised framework for reporting social media analytics research.

**Comment 9:**
3- Line 88, can you please confirm if the "this online tool" refers to Symplur?
**Reply:** Yes, it does, we have made it clearer in the paper.

**Comment 10:**
4- Line 108, 36% of user's demographics is presented in the paper, can you please confirm the location of the other users please?
**Reply:** Unfortunately, it is not possible to define the rest of the users’ locations as the majority of users’ locations are ‘unknown’. We have explained this more clearly in the update methods section.

**Comment 11:**
5- Line 110, please clarify what is meant by top users?
**Reply:** We have clarified this by including the definition of ‘top’:

“The top 20 Twitter users of #neoEBM, defined as the most frequent users of the hashtag,….”

**Comment 12:**
6- Line 112, can you please define the word bot, please?
**Reply:** A bot is a software application used to automatically generate messages, advocate ideas, act as a follower of users on social media platforms. We have elaborated on this definition when the term is introduced in the results section.

**Comment 13:**
7- Can you please comment on the total number of tweets if all of them were about neonatology? Was all the tweets were read/ a sample of the tweets were assessed as part of quality assurance to confirm that the tweets are about neonatology? Or if they were include based on face value, that all tweets were about neonatology? Or were there any user used the` hashtag to tweet about another topic?
**Reply:** Not all tweets were individually read but a word cloud highlights the content were universally related to neonatal practice (we can provide the word cloud if necessary). We can find no evidence that #NeoEBM is used by any other group or community

**Comment 14:**
8- Can you please confirm if there were any duplicated tweets?
**Reply:** There were no duplicated tweets – all were unique.

**Comment 15:**
9- Can you please provide the definition of the word engagement, please?
**Reply:** We are using the word engagement to mean “being involved with something (aka the CoP).” We have highlighted this in the revised paper by including the word “involvement” next to engagement to clarify its intended use in the paper.

**Comment 16:**
10- Have the team considered completing a content or thematic analysis of the tweets?
**Reply:** Yes, thank you for this suggestion. As this is significant piece of work, we think it would be better placed as a further piece of work.

**Comment 17:**
11- Can you please confirm if you had completed a word association thematic analysis, please?

**Reply:** Thank you for this query. No, we have not done this and would like to do this as an additional piece of work. Please see our response to the above.

**Comment 18:**
12- Can you please comment on the analysis that was completed using the software, Symplur, please?
**Reply:** Symplur is a twitter analytics platform which has developed trademarked algorithms to analyse content and also uses a taxonomy of 35000 terms mapped to 1 million social profiles which are then broken down into relevant healthcare stakeholders. Their software collates information around a specific hashtag and allows for deep content analysis via national language processing algorithms.

**Comment 19:**
13- Can you please add the information mentioned the price of the software in the method section, please?
**Reply:** We have added the following:

”We paid US$499 for access to the database for one month through the Symplur Signals Self-Serve (research option for 10 datasets) option.”

**Comment 20:**
Results
1- Line 120-121, can you please clarify the numbers please, as the numbers are not the same as the numbers presented in the abstract line 47-48. Can you please clarify if the numbers should add up to 3,228, please?
**Reply:** The number of users of #neoEBM across the study period was 3 228, this is consistent is the abstract and result sections.

To further clarify: “The majority of Twitter users made one tweet using #neoEBM (n=1843), followed by two tweets (n=443) and more than 10 tweets (n=347).” These numbers do not add up to 3 228 as some users made between 3-9 tweets and are not included in this breakdown of numbers.

We have corrected our error in the abstract and the main text. Thank you for picking this up.

**Comment 21:**
2- Can you please clarify the content of the tweets? What are the main themes within the tweets?
**Reply:** Please see our reply to Comment 13 (Reviewer 4) and previous comments about a thematic analysis.

**Comment 22:**
3- Can you please confirm if the tweets were used for information dissemination, increase awareness about certain topics, or networking? And what is the percentage of each component?
**Reply:** Thank you for this query. Unfortunately, it is not possible to draw inferences from the data regarding these points unfortunately due to the limitations of the third-party software currently available.

**Comment 23:**
4- What are the demographics of people tweeting using the hashtag? What was the gender of the people?
**Reply:** We have provided some of this data in the details regarding the top 20 users and in the Supplementary Material. Further, we have clarified in the methods that characteristics of users were obtained when publicly available in the profile.

**Comment 24:**
5- Can you please confirm what was the average engagement rate, please?
**Reply:** This was not formally calculated as part of this project. Our rationale was that as it is not part of the Aveling’s principles, and we did not formally calculate this.

**Comment 25:**
6- Can you please confirm if important information about the characterises of the tweets were factored in the analysis including, the length of the tweets, the time tweet was posted, the number of links in the tweets, and if these variables affected the engagement rate?

**Reply:** Thank you for these comments. hese factors were not factored into the analysis. Our rationale was that as it is not part of the Aveling’s principles, and we did not formally calculate this.

**Comment 26:**
Discussion
1- Line 160-161, can you please comment that this statement is based on an observation from 3 reply to one tweet, please?

**Reply:** Thank you for this comment. These statements were meant to highlight the potential criticisms of our work. Consequently, we have re-worded the sentence to hopefully make this more clear:

“Potential criticism of our work includes that it is no different to an email listserv, that it is at high risk of being overtaken by opinion-based medicine and that it has or will have no impact on clinical practice.”

6. PLOS authors have the option to publish the peer review history of their article ([what does this mean?](https://journals.plos.org/plosone/s/editorial-and-peer-review-process#loc-peer-review-history)). If published, this will include your full peer review and any attached files.

If you choose “no”, your identity will remain anonymous but your review may still be made public.

**Do you want your identity to be public for this peer review?** For information about this choice, including consent withdrawal, please see our [Privacy Policy](https://www.plos.org/privacy-policy).

Reviewer #1: No

Reviewer #2: No

Reviewer #3: No

Reviewer #4: No

[NOTE: If reviewer comments were submitted as an attachment file, they will be attached to this email and accessible via the submission site. Please log into your account, locate the manuscript record, and check for the action link "View Attachments". If this link does not appear, there are no attachment files.]

While revising your submission, please upload your figure files to the Preflight Analysis and Conversion Engine (PACE) digital diagnostic tool, <https://pacev2.apexcovantage.com/>. PACE helps ensure that figures meet PLOS requirements. To use PACE, you must first register as a user. Registration is free. Then, login and navigate to the UPLOAD tab, where you will find detailed instructions on how to use the tool. If you encounter any issues or have any questions when using PACE, please email PLOS at [figures@plos.org](mailto:figures@plos.org). Please note that Supporting Information files do not need this step.
